# High-cost high-need patients in Medicaid: segmenting the population eligible for a national complex case management program

**DOI:** 10.1186/s12913-021-07116-6

**Published:** 2021-10-23

**Authors:** Jacob K. Quinton, O. Kenrik Duru, Nicholas Jackson, Arseniy Vasilyev, Dennis Ross-Degnan, Donna L. O’Shea, Carol M. Mangione

**Affiliations:** 1 UCLA General Internal Medicine & Health Services Research, Los Angeles, California USA; 2 Department of Population Medicine, Harvard Medical School, Boston, Massachusetts USA; 3 United Healthgroup, Minnetonka, Minnesota USA

**Keywords:** Complex case management, Care management, High-cost high-need, Medicaid, Cluster analysis, Segmentation

## Abstract

**Background:**

High-cost high-need patients are typically defined by risk or cost thresholds which aggregate clinically diverse subgroups into a single ‘high-need high-cost’ designation. Programs have had limited success in reducing utilization or improving quality of care for high-cost high-need Medicaid patients, which may be due to the underlying clinical heterogeneity of patients meeting high-cost high-need designations.

**Methods:**

Our objective was to segment a population of high-cost high-need Medicaid patients (*N* = 676,161) eligible for a national complex case management program between January 2012 and May 2015 to disaggregate clinically diverse subgroups. Patients were eligible if they were in the top 5 % of annual spending among UnitedHealthcare Medicaid beneficiaries. We used *k*-means cluster analysis, identified clusters using an information-theoretic approach, and named clusters using the patients’ pattern of acute and chronic conditions. We assessed one-year overall and preventable hospitalizations, overall and preventable emergency department (ED) visits, and cluster stability.

**Results:**

Six clusters were identified which varied by utilization and stability. The characteristic condition patterns were: 1) pregnancy complications, 2) behavioral health, 3) relatively few conditions, 4) cardio-metabolic disease, and complex illness with relatively 5) low or 6) high resource use. The patients varied by cluster by average ED visits (2.3–11.3), hospitalizations (0.3–2.0), and cluster stability (32–91%).

**Conclusions:**

We concluded that disaggregating subgroups of high-cost high-need patients in a large multi-state Medicaid sample identified clinically distinct clusters of patients who may have unique clinical needs. Segmenting previously identified high-cost high-need populations thus may be a necessary strategy to improve the effectiveness of complex case management programs in Medicaid.

**Supplementary Information:**

The online version contains supplementary material available at 10.1186/s12913-021-07116-6.

## Background

Rising costs in Medicaid are a key threat to balanced budget requirements in US states, enhancing pressure on Medicaid administrators to control costs, especially during periods of economic recession [1]. The majority of expenditure in any insured group concentrates in a few patients, with 5% of individuals accounting for approximately 50% of expenditure year-to-year [2]. The stability of this cost distribution has led to an actuarily important designation of patients as high-cost high-need, although defining groups by cost or risk thresholds aggregates clinically distinct subgroups and definitions for high-cost high-need vary [3]. There is also substantial regression to the mean of the cost distribution among individuals in this grouping of outliers [4]. High-cost high-need patients have been disaggregated into clinically distinct subgroups or ‘segmented’ at various cost thresholds in many risk-bearing practice settings [5] and integrated health systems [6, 7], as well as at the population level by insurance risk pool including Medicare [8] and Medicare Advantage [9], with the results being incorporated into a national model of high-cost high-need patients [10] and informing value-based payment models in the Medicare program [11].

Preliminary descriptions of high-cost high-need patients in Medicaid have shown aggregation by cost thresholds has led to clinically heterogeneous high-cost high-need populations—some with multiple co-morbidities [12], substance use disorder, or serious mental illness [13], and many with unmet health-related social needs possibly contributing to their use of health care [14]. The paucity of multi-state or national Medicaid high-cost high-need segmentation analyses may not just be overlooking opportunities to test value-based payment models, but also may be exacerbating disparities when using uniform complex case management programs. Complex case management programs, frequently phone-based care coordination programs led by RN case managers [15], have been shown to be effective in Medicare [16] but have been difficult to effectively implement in Medicaid, leading to notable high profile programs with null findings [17], and some small positive demonstrations [18].

The objective of our study is to disaggregate or segment a multi-state sample of high-cost high-need Medicaid patients who were eligible for a national complex case management program led by UnitedHealthcare to address medical, behavioral, and unmet social needs through a phone-based RN-led complex case management intervention, which was evaluated previously by our research team and found to be ineffective at reducing hospitalizations or emergency room visits in most Medicaid beneficiaries [19].

## Methods

### Study population

Our beginning sample consisted of 676,161 Medicaid beneficiaries age 21 and above who were identified as eligible for a complex case management program implemented by UnitedHealthcare (UHC) in 15 states between Jan 1, 2012 and May 1, 2015. Individuals with one full year of observation before enrollment in the program were included in our analytic sample (*N* = 484,328) and our sample included only the year of pre-enrollment observation time. This study is not evaluating the complex case management program for which they were eligible. UHC’s criteria used to identify eligible individuals were: 1) patients in the top 5% of spending the prior year, and 2) UHC’s proprietary risk score identifying patients likely to persist in the top 5% of spending in the following year. This risk score has been internally validated by UHC and is used across their enterprise. UCLA authors are blinded to all score components as well as the derivation and validation of risk score.

### Data

Data available for analysis included demographic information, medical, pharmacy, and laboratory claims, and unmet social need and barriers to care data for a subset of patients. We include demographic information as well as all medical (including behavioral health) claims. Acute and chronic conditions were assessed from ICD-9-CM codes for all claims in the observation period and grouped according to the Agency for Healthcare Research and Quality (AHRQ) multi-level Clinical Classification Software (CCS) for ICD-9-CM [20]. Utilization was not used to define clusters despite demonstrated variation in high-cost high-need patients’ utilization trajectories [21] to allow for unbiased comparisons between clusters. Preventable hospitalizations were assessed as conditions considered possibly preventable in the AHRQ prevention quality indicators [22] and preventable ED visits were assessed using the NYU/Billings algorithm for assessing possibly preventable ED utilization [23].

### Choice of partitional clustering and k-means

Statistical learning can be divided into supervised and unsupervised methods, where supervised methods such as regression and classification have a set of features and a response measured on these features is predicted. Unsupervised learning methods such as principal component analysis and cluster analysis are a set of statistical tools intended for when there is no response, and the goal is to observe patterns in the set of features, such as the clinical subgroups aggregated within a high-cost high-need population, not prediction [24]. Cluster analyses are a broad set of techniques to find subgroups or clusters in a data set, and are defined as a special case of a normal mixture model which assumes that the mixture components (i.e., clusters) have spherical covariance matrices (i.e., equal variance) and equal sampling probabilities [25]. Partitional clustering techniques (like *k-*means) assign all observations (n) to one of a number of pre-specified subgroups (*k*). All observations must be assigned, and the subgroups are non-overlapping. The *k*-means algorithm begins by randomly assigning each observation to one of *‘k’* clusters in n*-*dimensional space, and then calculates the centroid (i.e., mean position) of the cluster. It iterates by reassigning the closest observations to the previously calculated centroid, then recalculating the centroid. This proceeds until the cluster assignment is stable. We chose *k*-means partitional clustering as a robust and computationally efficient technique, which was important given the large sample size.

### Number of clusters and mixed data

There is no obvious way to determine the optimal number of subgroups in partitional cluster analysis, and techniques to accomplish this are an active area of investigation [24]. In our approach we used an information-theoretic or ‘jump’ method to identify the number of clusters where the improvement in explanatory power (or reduction in error) of the clusters ‘jumped’ the most when we increased the number of clusters from *k* to *k* + 1 [26]. This is measured by the change in the test statistic of interest (partial F-test statistic) which decreases monotonically when adding an additional cluster. To determine the optimal number of clusters we pre-specified clusters (*k*) between 2 and 20 and plotted the partial F statistic by cluster number (*k*). Modifications to cluster analyses allowing for mixed datasets are another ongoing area of investigation to allow for clusters to be developed using both continuous and categorical data features [27]. As the clustering proceeds in *n-*dimensional space, it is not intuitive how categorical variables would be arrayed in space. To account for categorical data features, we transformed all demographic and condition categorical variables to polar coordinates, as this was a high performing approach compared to other transformations and was also computationally efficient [28].

### Cluster stability

Cluster analyses are unsupervised methods and as such variation in the data features determines cluster assignment. Partitional clustering forces all observations to one of a prespecified number of clusters. Given all observations are forced into a cluster, there is the need to verify that observations are reliably assigned to similar clusters when sampling from similar datasets. This ‘cluster stability’ is an important robustness check. We resampled with replacement (bootstrapped) a large number of replications (*n* = 500) and identified cluster assignments for each iteration. A limitation to the k-means protocol in SAS is the ordering of the clusters is random (i.e. cluster ‘1’ in the original analysis may be labeled cluster ‘3’ in one of the bootstrapped iterations). To overcome this, we reassigned cluster numbers based on the highest interrater reliability (kappa) between the original cluster and the bootstrapped clusters.

There are many indexes of cluster stability, and we chose two commonly used measures. Each index compares one set of observations with another set (i.e. our ‘original’ sample and the 500 bootstrapped iterations) and compares dissimilarity. The Rand Index (or % observed agreement) represents the percentage of time the original cluster assignment agreed with the bootstrapped assignment for all possible comparisons. The Jaccard coefficient (or % overlap) measures only the times when the same assignment was made as a proportion of the total times the cluster was assigned in either the original or bootstrap sample. The observed agreement (Rand) is thus an easily identifiable measure of cluster similarity, and the percent overlap (Jaccard) as a more sensitive measure of cluster similarity [29]. As these are uncommon measures, we explain the cluster stability indexes more in supplementary materials (Additional file 2).

### Cluster labeling

We used demographic information (age, gender, Medicaid program of eligibility, race/ethnicity, language) and diagnostic groupings using the AHRQ CCS multi-level categories (at the 2nd level) as inputs to the *k*-means partitional clustering algorithm. To create a clinically meaningful label for each cluster we examined the descriptive statistics for our cluster inputs (e.g. demographics and condition categories) and identified the features of the cluster that that maximally deviated from the overall mean. Through discussion among the investigators, we identified cluster labels that were both representative of the pattern of outliers and reasonable to clinicians.

## Results

### Demographics and diagnostic categories

**Fig. 1:**
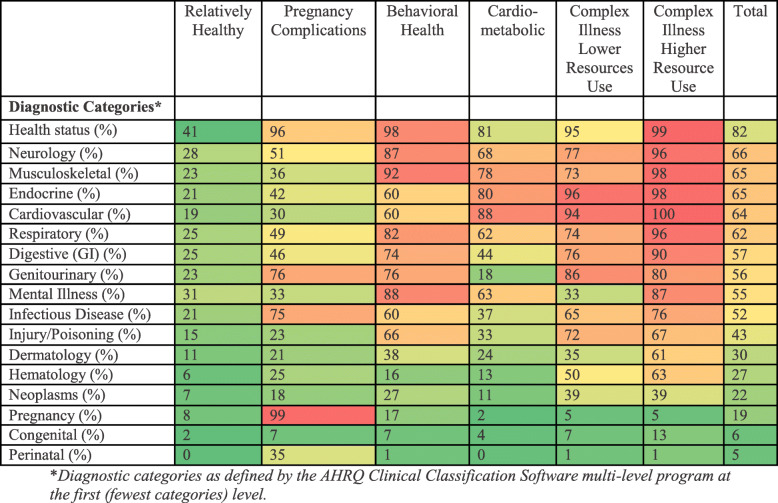
Heat map of acute and chronic condition categories by cluster for high-cost high-need Medicaid patients eligible for national complex case management program

We determined the optimal number of clusters to be six, and partitioning of observations was non-overlapping and varied (13–21%) between clusters (Table 1). Demographics, including average age and gender, and program of Medicaid eligibility varied by cluster (Table 1). Acute and chronic conditions varied widely by cluster, and these were the primary features we used for assigning a descriptive label to each cluster, and these we displayed as a heat map (Fig. 1). Cluster 1 had a pattern of relatively fewer diagnoses, so we labeled it ‘Relatively Healthy’. Nearly all (98%) individuals in the second cluster were diagnosed with a complication of pregnancy so we labeled this cluster ‘Pregnancy Complications’. The highest percent of individuals with mental illness (90%) and second highest rate of neurologic disorders (86%) were seen in the 3rd cluster, so we labeled it ‘Behavioral Health’. The 4th cluster, despite having a relatively low prevalence of most diagnoses, had the second highest rates of endocrine (96%) and cardiovascular (93%) disorders and so we labeled it labeled ‘Cardiometabolic’. The remaining groups had similar frequencies of diagnoses across a large number of diagnostic categories but very different utilization patterns and so we labeled them ‘Complex Illness Lower Resource Use’ and ‘Complex Illness Higher Resource Use’ to reflect these differences in utilization but shared medical complexity.

**Table 1:** Demographic information by cluster for high-cost high-need Medicaid patients eligible for national complex case management program

	Relatively Healthy	Pregnancy Complications	Behavioral Health	Cardio-metabolic	Complex Illness Lower Resource Use	Complex Illness Higher Resource Use	Total
**Population**	100,247	63,329	80,760	94,135	77,871	67,985	484,328
**Demographics**							
Age (mean)	42	28	40	51	54	52	45
Female (%)	61	100	84	45	68	65	69
Medicaid eligibility (%)							
TANF	58	95	58	34	40	26	52
SSI	34	4	35	54	47	61	39
Expansion	6	1	6	8	6	6	6
Medicare-eligible	2	< 1	1	4	6	7	3
Race/Ethnicity (%)							
White	50	48	62	50	38	49	50
Black	23	27	17	21	21	18	21
Hispanic	6	9	5	5	10	5	7
Asian	3	4	1	4	8	2	4
Other	2	1	2	1	2	1	1
Missing	16	11	13	19	21	24	17

### Utilization and preventable utilization

One-year mean utilization varied by cluster, including hospital admissions (0.3–2.0 admissions), days in hospital (2.1–16.0 days), and ED visits (2.3–11.3 visits). Preventable utilization varied by cluster for mean preventable hospitalizations (0.01–0.33 admissions) and mean preventable ED visits (1.1–6.3 visits). Mean hospital admissions in the major complex illness cluster were six times higher than in any other cluster (Table 2). Mean ED visits were highest in the complex illness higher resource use cluster and second highest in the behavioral health cluster.

**Table 2:** Utilization and preventable utilization by cluster for high-cost high-need Medicaid patients eligible for national complex case management program

	Relatively Healthy	Pregnancy Complications	Behavioral Health	Cardio-metabolic	Complex Illness Lower Resource Use	Complex Illness Higher Resource Use	Total
Hospital Admissions Mean (SD)	0.3 (2.3)	0.8 (0.6)	0.4 (1.2)	0.4 (1.7)	0.5 (1.5)	2.0 (3.3)	0.7 (2.0)
Hospital Days Mean (SD)	4.3 (35.4)	2.5 (4.2)	2.1 (58.1)	2.8 (22.7)	3.2 (21.2)	16.0 (39.0)	4.9 (35.1)
ED visits Mean (SD)	2.9 (6.1)	4.7 (6.2)	7.9 (12.6)	4.8 (8.5)	4.9 (8.4)	14.6 (26.7)	7.5 (15.8)
Preventable Hospitalizations Mean (SD)	0.01 (0.08)	0.02 (0.13)	0.04 (0.20)	0.06 (0.24)	0.10 (0.30)	0.33 (0.47)	0.09 (0.28)
Preventable ED visits Mean (SD)	1.1 (1.7)	1.9 (2.3)	2.9 (4.0)	1.9 (2.7)	2.4 (3.1)	6.3 (10.0)	3.1 (5.7)

### Cluster stability

Cluster stability was assessed through repeated resampling with replacement and stability determined by percent agreement with the original cluster assignment (Table 3). There was a gradient of cluster stability as defined by the Jaccard coefficient from less stable to very stable (32–91%). For each cluster we report the mean of the Jaccard and Rand coefficients, respectively. Two of the six clusters, the cluster of relatively healthy patients (84, 96%) and of women experiencing complications of pregnancy (91, 99%) were very stable. The clusters of patients experiencing utilization due to behavioral health needs (48, 88%) and complex illness with higher resource use (64, 94%) were fairly stable, and the remaining two clusters, one of patients with cardiometabolic disease (32, 81%) and complex illness with lower resource use (42, 86%) were less stable.

**Table 3:** Cluster Stability by Cluster

	Relatively Healthy	Pregnancy Complications	Behavioral Health	Cardio-metabolic	Complex Illness Lower Resource Use	Complex Illness Higher Resource Use	Total
**Rand Index (%)**							
Mean (SD)	96 (1)	99 (3)	88 (5)	81 (7)	86 (5)	94 (2)	90 (4)
**Jaccard Coefficient (%)**							
Mean (SD)	84 (5)	91 (11)	48 (12)	32 (19)	42 (15)	64 (12)	59 (12)

## Discussion

High-cost high-need patients as defined by a risk or cost threshold aggregate clinically diverse patient subgroups, and complex case management programs may be improved by first disaggregating or segmenting the underlying high-cost high-need population. We found in a large, multi-state sample of high-cost high-need Medicaid patients six clusters which varied by demographics, utilization, and preventable utilization. Consistent with our hypothesis clinically distinct clusters such as women experiencing complications of pregnancy or patients with utilization related to behavioral health concerns emerged from the data and may be appropriate targets for specialized complex case management programs in Medicaid. Interestingly, these clinically distinct clusters were also some of the most stable, reinforcing their utility as possible targets for complex case management programs.

Cluster stability measures are important for considering how small changes in the underlying population may affect the emergent subgroups. Cluster assignment in our analysis was primarily driven by the pattern of acute and chronic conditions, and thus, the large number of high-cost high-need patients with multiple chronic conditions were more challenging to separate from others and we observed these clusters to be the least stable. This does not necessarily mean complex case management programs designed to manage patients with multiple chronic conditions will be ineffective—to the contrary, most current programs target this population. The stability measures are more important for identifying unique and stable subpopulations previously unobservable in the aggregated high-cost high-need population. It is intuitive that women experiencing complications of pregnancy and patients seeking care for behavioral health needs have different care coordination needs than patients whose care is defined by complex illness. We also found a less stable cluster of patients defined by cardiometabolic disease, which is not to say patients with these conditions are not likely to be found in high-cost high-need Medicaid populations, only that in this population patients with cardiometabolic disease frequently had other conditions which caused them to be challenging to distinguish from patients with complex illness. Finally, the relatively stable group of high-cost high need patients who were using relatively lower levels of care warrant further investigation. Perhaps they are patients whose need for medical care has regressed to the mean. This cluster may yet have their own needs for complex case management as yet unidentified by our study.

Our study has several limitations. Although our sample consists of a large multi-state Medicaid population from a major health plan, small states or individual health systems may have unique clusters that don’t persist in a large, multi-state sample. Also, because our team had data only for those eligible for the complex case management program, these individuals may differ systematically from the overall population in ways we didn’t anticipate.

Moving forward, we hope this work fosters continued innovation in population health management. It provides a framework that can be used by state Medicaid agencies and Medicaid managed care organizations to segment their high-cost high-need populations to determine how best to tailor their population health strategy. Once administrators disaggregate stable subgroups, the next step is to tailor complex case management to the unique clinical needs of the subgroup. There has been substantial work done in this area in Medicaid, including outpatient extensivist teams for patients with behavioral health conditions [30], qualitative work addressing the needs of pregnant women utilizing unscheduled care [31], disease-focused complex case management [19], and more intensive home-based interventions utilizing community health workers [18, 32]. Finally, the increasing availability of national systematic samples may facilitate developing a generalizable model of high-cost high-need patient subgroups in Medicaid which would speed the development of tools to identify patients appropriate for complex case management or other intervention strategies.

## Conclusion

We found that using an unsupervised approach (cluster analysis) we were able to reliably disaggregate a high-cost high-need population of Medicaid patients into clinically distinct subgroups. These subgroups may benefit from targeted complex case management programs. We encourage state Medicaid administrators as well as managed Medicaid health plan leaders to consider segmentation as a strategy to identify distinct subgroups within a heterogeneous high-cost high-need population.

## Supplementary Information

**Additional file 1: Appendix 1.** Annotated code to run *k-*means clustering protocol. Code for SAS 9.4.

**Additional file 2: Appendix 2.** Cluster Stability Indexes.

## Data Availability

The datasets analyzed were raw medical and eligibility claims from UnitedHealthcare and are not publicly available, and are not available due to the requirements of the data use agreement between UCLA researchers and United Healthgroup, but deidentified variables that are a byproduct of the analysis are available from the corresponding author and UnitedHealthcare on reasonable request.
