# Supplementary material for: High-cost high-need patients in Medicaid: segmenting the population eligible for a national complex case management program
Source: BMC Health Serv Res. 2021 Oct 23;21:1143. doi: 10.1186/s12913-021-07116-6 (PMC8539737; doi:10.1186/s12913-021-07116-6)
Supplement: Supplementary file 1 — Additional file 1: Appendix 1. Annotated code to run k-means clustering protocol. Code for SAS 9.4. [file 12913_2021_7116_MOESM1_ESM.docx]

**Appendix 1**: Annotated code to run *k-*means clustering protocol. Code for SAS 9.4

/* **Step 1 Notes**: Clustering with mixed data features: Clustering algorithms separate each input data feature along multi-dimensional space. This works well for continuous variables that are standardized to a minimum value of -1, maximum value of 1. This works reasonably well for ordinal variables, and does not work well for categorical variables. */

/* **Step 1**: Standardizing data features */

**/* Step 1 Example**:

mval_varname = X;

max_varname = Y;

mcenter_varname = mval_varname - varname;

abs_varname=abs(mcenter_varname);

std_varname = (varname - mval_varname)/max_varname;

*/

/***Step 1 hardcoded standardizing age variable** */

mval_age = 42.6; /* this is the mean age value for all observations */

max_age = 112; /* this is the maximum value for all observations */

mcenter_age = mval_age - age;

abs_age = abs(mcenter_age);

std_age = (age - mval_age)/max_age;

/* **Step 2 Notes**: Once variables are standardized, recent literature suggests transforming to polar or spherical coordinates as the optimal management for categorical variables. We chose polar coordinates for simplicity. In the linked article this equation is given by Cati = [cos((i-1)*2*pi)/N], [sin((i-1)*2*pi)/N] where i is the category number and N is the total number of categories. A quote from the below linked article “An exception for the conversion of one categorical input into 2 numerical ones is when the categorical input is binary. In that case there is no need for 2 new coordinates but for one, given that the division of the circle into 2 points will have both of them projected onto the same axis and then the other coordinate is in both cases null and can be removed.” [F. Barcelo-Rico and J.L. Diez, “Geometrical codification for clustering mixed categorical and numerical databases,” J. Intell. Inf. Syst., vol. 39, no. 1, pp 167-185, 2012.] Most of our data features are binary, and as such have only one value (such as all ccs values).

/* ccs1 */

if ccs1 = 1 then ccs1x=-1; else ccs1x = 1;

/* **Step 2 Notes continued**: When the data is more than binary, two points (x, y) must be specified, these will always have the same values based on the number of total categories (N).

/* lang_cat4 */

if lang_cat4 = 1 then lang_cat4x = 1;

if lang_cat4 = 1 then lang_cat4y = 0;

if lang_cat4 = 2 then lang_cat4x = 0;

if lang_cat4 = 2 then lang_cat4y = 1;

if lang_cat4 = 3 then lang_cat4x = -1;

if lang_cat4 = 3 then lang_cat4y = 0;

if lang_cat4 = 4 then lang_cat4x = 0;

if lang_cat4 = 4 then lang_cat4y = -1;

/* race_eth */

if race_eth = 1 then race_ethx = 1;

if race_eth = 1 then race_ethy = 0;

if race_eth = 2 then race_ethx = 0.623504021;

if race_eth = 2 then race_ethy = 0.781820143;

if race_eth = 3 then race_ethx = -0.222485472;

if race_eth = 3 then race_ethy = 0.974936005;

if race_eth = 4 then race_ethx = -0.900945194;

if race_eth = 4 then race_ethy = 0.433932896;

if race_eth = 5 then race_ethx = -0.901000429;

if race_eth = 5 then race_ethy = -0.433818195;

if race_eth = 6 then race_ethx = -0.222609587;

if race_eth = 6 then race_ethy = -0.974907673;

if race_eth = 7 then race_ethx = 0.623404484;

if race_eth = 7 then race_ethy = -0.781899513;

/* **Step 3:** Once data is standardized and categorical features are dealt with by transitioning to a coordinate set, the SAS procedure can be used. This example uses standardized age, race/ethnicity categories, and the acute and chronic condition categories used (hardcoded, any of the AHRQ CCS categories that were null or blank for our data were removed). The maximum iterations for which the algorithm will seek local minima are specified, and the maximum clusters ‘maxclusters’ is prespecified (here the number is 6).

PROC FASTCLUS DATA=d.pre_cco_data_agerest_std out=d.pre_cco_data_agerest_std_clus maxclusters=6 maxiter=100;

var std_age race_ethx race_ethy

ccs1x ccs2x ccs3x ccs4x ccs5x ccs6x ccs11x ccs12x ccs13x ccs14x ccs15x ccs16x ccs17x ccs18x ccs19x ccs20x ccs21x ccs22x ccs23x ccs24x ccs25x ccs26x ccs27x ccs28x ccs29x ccs30x ccs31x ccs32x ccs33x ccs34x ccs35x ccs36x ccs37x ccs38x ccs39x ccs40x ccs41x ccs42x ccs43x ccs44x ccs45x ccs46x ccs47x ccs48x ccs49x ccs50x ccs51x ccs52x ccs53x ccs54x ccs55x ccs56x ccs57x ccs58x ccs59x ccs60x ccs61x ccs62x ccs63x ccs64x ccs65x ccs66x ccs67x ccs68x ccs69x ccs70x ccs71x ccs72x ccs73x ccs74x ccs75x ccs76x ccs77x ccs94x ccs95x ccs96x ccs97x ccs98x ccs99x ccs100x ccs101x ccs102x ccs103x ccs104x ccs105x ccs106x ccs107x ccs108x ccs109x ccs110x ccs111x ccs112x ccs113x ccs114x ccs115x ccs116x ccs136x ccs137x ccs138x ccs139x ccs148x ccs149x ccs150x ccs151x ccs152x ccs153x ccs154x ccs155x ccs156x ccs157x ccs158x ccs159x ccs160x ccs163x ccs170x ccs171x ccs172x ccs173x ccs174x ccs175x ccs176x ccs177x ccs178x ccs179x ccs181x ccs182x ccs183x ccs184x ccs185x ccs186x ccs199x ccs200x ccs201x ccs202x ccs203x ccs204x ccs205x ccs206x ccs209x ccs210x ccs211x ccs212x ccs213x ccs214x ccs215x ccs216x ccs217x ccs218x ccs219x ccs220x ccs221x ccs252x ccs253x ccs254x ccs255x;

RUN;

/* **Step 4**: This procedure produces your cluster assignments, however visualizing the data can be nice. To do this you can obtain the canonical variable (reducing the multi-dimensional output to two dimensions) analysis as a visual check. This is a form of principal component analysis. We used these during our analysis but did not include the figures in the final manuscript.*/

PROC CANDISC DATA=d.pre_cco_data_std_clus out=d.pre_cco_data_std_clus_can noprint;

class cluster;

var std_age

femalex

race_ethx race_ethy

ccs1x ccs2x ccs3x ccs4x ccs5x ccs6x ccs11x ccs12x ccs13x ccs14x ccs15x ccs16x ccs17x ccs18x ccs19x ccs20x ccs21x ccs22x ccs23x ccs24x ccs25x ccs26x ccs27x ccs28x ccs29x ccs30x ccs31x ccs32x ccs33x ccs34x ccs35x ccs36x ccs37x ccs38x ccs39x ccs40x ccs41x ccs42x ccs43x ccs44x ccs45x ccs46x ccs47x ccs48x ccs49x ccs50x ccs51x ccs52x ccs53x ccs54x ccs55x ccs56x ccs57x ccs58x ccs59x ccs60x ccs61x ccs62x ccs63x ccs64x ccs65x ccs66x ccs67x ccs68x ccs69x ccs70x ccs71x ccs72x ccs73x ccs74x ccs75x ccs76x ccs77x ccs94x ccs95x ccs96x ccs97x ccs98x ccs99x ccs100x ccs101x ccs102x ccs103x ccs104x ccs105x ccs106x ccs107x ccs108x ccs109x ccs110x ccs111x ccs112x ccs113x ccs114x ccs115x ccs116x ccs136x ccs137x ccs138x ccs139x ccs148x ccs149x ccs150x ccs151x ccs152x ccs153x ccs154x ccs155x ccs156x ccs157x ccs158x ccs159x ccs160x ccs163x ccs170x ccs171x ccs172x ccs173x ccs174x ccs175x ccs176x ccs177x ccs178x ccs179x ccs181x ccs182x ccs183x ccs184x ccs185x ccs186x ccs199x ccs200x ccs201x ccs202x ccs203x ccs204x ccs205x ccs206x ccs209x ccs210x ccs211x ccs212x ccs213x ccs214x ccs215x ccs216x ccs217x ccs218x ccs219x ccs220x ccs221x ccs252x ccs253x ccs254x ccs255x;

RUN;

PROC SGPLOT DATA=pre_cco_data_std_clus_can;

scatter y=can2 x=can1 / group=cluster;

RUN;

quit;
