# Supplementary material for: High-cost high-need patients in Medicaid: segmenting the population eligible for a national complex case management program
Source: BMC Health Serv Res. 2021 Oct 23;21:1143. doi: 10.1186/s12913-021-07116-6 (PMC8539737; doi:10.1186/s12913-021-07116-6)
Supplement: Supplementary file 2 — Additional file 2: Appendix 2. Cluster Stability Indexes. [file 12913_2021_7116_MOESM2_ESM.docx]

**Appendix 2: Cluster Stability Indexes**

We chose in our analysis two commonly used measures of cluster stability. One of the most common questions in presenting this work is to ask for more details regarding cluster stability measures as this is an uncommon topic.

The Rand Index (or % observed agreement) represents the percentage of time the original cluster assignment agreed with the bootstrapped assignment for all possible comparisons (i.e. the number of times an observation assigned to cluster ‘1’ in the original dataset was assigned to cluster ‘1’ in the comparison as well as the number of times an observation was assigned to other clusters in both the original and comparison datasets, over the total number of times that observation assigned to cluster ‘1’ was assigned to any cluster in the comparison dataset). Using the 2 x 2 tables familiar from epidemiology, the Rand Index includes true positives (TP) and true negatives (TN) in the numerator divided by the sum of TP, false positives (FP), false negatives (FN) and TN in the denominator.

$$Rand Index=\frac{TP+TN}{TP+FN+FP+TN}$$

The Jaccard coefficient (or % overlap) measures only the times when the same assignment was made as a proportion of the total times the cluster was assigned in either the original or bootstrap sample (i.e. the number of times an observation assigned to cluster ‘1’ in the original dataset was assigned to cluster ‘1’ in the comparison, and the times an observation was assigned to cluster ‘1’ in both datasets over the total number of times that observation assigned to cluster ‘1’ in either the comparison or original dataset, including when it was assigned to ‘1’ in both datasets). Taking the 2 x 2 tables again, the Jaccard index is TP divided by FP + FN + TP.

$$Jaccard Coefficient=\frac{TP}{TP+FN+FP}$$

Put this way, while there is a commonly explainable measure (observed agreement) for the Rand index, the total number of comparisons will be very different depending on the total number of options (i.e. if there were four or five clusters in the analysis, there would be very different ‘TN’ numbers). Thus the Jaccard coefficient is more directly comparable and more sensitive (as the percent overlap).
